# Editorial: *C. elegans* MAPS the way: the 2022 *C. elegans* meeting on metabolism, aging, pathogenesis, and stress (MAPS 2022)

**DOI:** 10.3389/fphys.2023.1331912

**Published:** 2023-11-13

**Authors:** Lesley MacNeil, Jian Li

**Affiliations:** ^1^ Department of Biochemistry and Biomedical Sciences, McMaster University, Hamilton, ON, Canada; ^2^ New York Medical College, Valhalla, NY, United States

**Keywords:** *C. elegans*, MAPS meeting, metabolism, aging, stress, pathogen, conference summary

The Metabolism, Aging, Pathogenesis and Stress in *C. elegans* (MAPS) conference took place at the University of Wisconsin, Madison July 14-17, 2022. This bi-yearly meeting gathers researchers from around the world who use *C. elegans* to answer fundamental physiological questions. The advantage of *C. elegans* as a model lies in both its simplicity and complexity. Its small size, short life cycle, simple body plan and ease of manipulation enable analyses not feasible with more complex animal models. While the ease of use is on par with cell-based assays, the complexity of *C. elegans*, relative to cell-based assays, is invaluable in studying many important biological questions at the organismal level, including Metabolism, Aging, Pathogenesis, and Stress Response (MAPS). This Research Topic provides a collection of articles highlighting the topics that were the focus of this conference.

Thanks to its short lifespan and powerful genetics, *C. elegans* is a widely used model for aging and its use has led to many ground-breaking discoveries. One prominent mechanism of longevity is robust stress responses to environmental perturbation and pathogens. Increasing evidence suggests that animal stress responses are subject to both cell-autonomous and non-autonomous regulation. The simple body plan and fully mapped cell lineage of *C. elegans* provide unique opportunities to understand inter-tissue stress signaling. In this research topic, Oosten-Hawle reviews the latest research on stress response pathways that preserve proteostasis and their regulation by inter-tissue signals. Since the loss of proteostasis is a hallmark of aging, understanding how proteotoxic stress responses are orchestrated at the organismal level has particular significance in aging studies. At the molecular level, stress-responsive transcription factors play critical roles in maintaining homeostasis. Doering et al. review the contributions of one such transcription factor, NHR-49 in regulating metabolism and aging. They summarize the current knowledge of regulatory inputs and tissue-specific transcriptional outputs of NHR-49 in stress responses and innate immunity and describe how these functions impact healthy aging. The final review in the Research Topic, a summary of current knowledge about glucosylceramides in *C. elegans* by Xatse and Olsen, describes the synthesis and degradation of these molecules and their potential roles as signaling molecules and structural components.

*C. elegans* are bacterivores. In their natural environment, worms are exposed to a plethora of both beneficial and pathogenic bacteria to which they must respond appropriately. This feature makes *C. elegans* an excellent model for studying host-microbe interactions that are relevant to the actions of the microbiome and bacterial pathogens. Since Sydney’s Brenner’s introduction of the worm as a model system, we have used the uracil auxotroph, *E. coli* OP50, as the main laboratory *C. elegans* food source. This strain was chosen not because of its nutritional qualities, but because the growth-limited lawn facilitates visualization of worms. Consequently, much of what we have learned about phenotypes has been achieved under a single environmental condition. Work from several groups has shown that different bacterial species and strains can have profound effects on phenotype. In this Research Topic, Stover et al. describe how the lactic acid bacteria *Lactococcus lactis* and *Leuconostoc mesenteroides* increased lifespan when added to the standard *E*. *coli* OP50 diet. Lactic acid bacteria are commonly used as probiotics and are generally believed to have beneficial health effects. To understand the survival-promoting properties of the bacteria, the authors measured basal stress levels and stress response in animals fed diets composed of individual or mixed bacteria. They measured the heat shock response and unfolded protein responses of the endoplasmic reticulum and mitochondria, finding that different bacterial diets induce different patterns of stress response. Future genetic studies will be needed to examine the causal relationship between altered stress responses and increased lifespan and test the contributions of other longevity pathways upon supplementing the lactic acid bacteria.

Transgenerational effects of diet and pathogen exposure were also explored at the MAPS meeting. In this Research Topic, Wibisono and Sun examined transgenerational effects of pathogen exposure on gene expression and fitness. Training animals with *S enterica* over several generations did not alter survival in subsequent generations but did decrease survival variability between generations. When examining the survival of animals exposed to the opportunistic pathogen *Salmonella enterica* in both unexposed and primed lineage for 12 generations, they discovered reproducible patterns of survival and expression of *lipl-1,* an enzyme of the TFEB lipophagic pathway. The authors hypothesized that a previously unaccounted-for epigenetic phenomenon may induce random adjustments to gene expression in untrained animals that underlie the observed transgenerational patterns.

With the expansion of our knowledge of physiology in *C. elegans* comes the need to expand the *C. elegans* toolkit in this area. One challenge in *C. elegans* aging research is the requirement to either prevent the production of offspring or continuously transfer adults to new plates for lifespan assays. Temperature-sensitive sterile animals or the addition of FUDR have been used to prevent offspring. However, each of these approaches can introduce complications, highlighting the need for additional tools. Beydoun et al. examined one such potential tool, C22, a small molecule that impairs eggshell integrity and disrupts early embryogenesis in lifespan. They found C22 could extend lifespan, likely through induction of FMO-2, a longevity-regulating enzyme that acts downstream of dietary restriction. While further investigation is required to elucidate the exact mechanisms underlying C22 mediated longevity, it calls for caution in using C22 in lifespan analysis.

